# Halloysite Nanotubes and Silane-Treated Alumina Trihydrate Hybrid Flame Retardant System for High-Performance Cable Insulation

**DOI:** 10.3390/polym13132134

**Published:** 2021-06-29

**Authors:** Sandra Paszkiewicz, Izabela Irska, Iman Taraghi, Elżbieta Piesowicz, Jakub Sieminski, Karolina Zawisza, Krzysztof Pypeć, Renata Dobrzynska, Agnieszka Terelak-Tymczyna, Kamil Stateczny, Bartłomiej Szymczak

**Affiliations:** 1 Department of Materials Technology, West Pomeranian University of Technology, Piastow Av. 19, PL-70310 Szczecin, Poland; izabela.irska@zut.edu.pl (I.I.); taraghi.iman@gmail.com (I.T.); elzbieta.piesowicz@zut.edu.pl (E.P.); jakub.sieminski@tfkable.com (J.S.); kpypec@zut.edu.pl (K.P.); 2 TELE-FONIKA Kable S.A., Cegielskiego 1, PL-32400 Myślenice, Poland; karolina.zawisza@tfkable.com; 3 STARGUM Zaklad Przemyslu Gumowego, Cieplna 1, PL-73100 Stargard, Poland; 4 Materials Fire Properties Testing Laboratory, West Pomeranian University of Technology, Piastow Av. 41, PL-71065 Szczecin, Poland; renata.dobrzynska@zut.edu.pl; 5 Department of Production Management, West Pomeranian University of Technology, Piastow Av. 19, PL-70310 Szczecin, Poland; agnieszka.terelak@zut.edu.pl (A.T.-T.); kamil.stateczny@zut.edu.pl (K.S.); 6 Department of Mechatronics, West Pomeranian University of Technology, Piastow Av. 19, PL-70310 Szczecin, Poland; bartlomiej.szymczak@zut.edu.pl

**Keywords:** flame retardation of polymers, green flame retardants for polymers, polymers for electrical cables, cable insulation, halloysite nanotubes, silane-treated ATH, morphology, mechanical performance, thermal stability

## Abstract

The effect of the presence of halloysite nanotubes (HNTs) and silane-treated alumina trihydrate (ATH-sil) nanofillers on the mechanical, thermal, and flame retardancy properties of ethylene-vinyl acetate (EVA) copolymer/low-density polyethylene (LDPE) blends was investigated. Different weight percentages of HNT and ATH-sil nanoparticles, as well as the hybrid system of those nanofillers, were melt mixed with the polymer blend (reference sample) using a twin-screw extruder. The morphology of the nanoparticles and polymer compositions was studied using scanning electron microscopy (SEM) and energy dispersive spectroscopy (EDS). The mechanical properties, hardness, water absorption, and melt flow index (MFI) of the compositions were assessed. The tensile strength increases as a function of the amount of HNT nanofiller; however, the elongation at break decreases. In the case of the hybrid system of nanofillers, the compositions showed superior mechanical properties. The thermal properties of the reference sample and those of the corresponding sample with nanofiller blends were studied using differential scanning calorimetry (DSC) and thermogravimetric analysis (TGA). Two peaks were observed in the melting and crystallization temperatures. This shows that the EVA/LDPE is an immiscible polymer blend. The thermal stability of the blends was improved by the presence of HNTs and ATH-sil nanoparticles. Thermal degradation temperatures were shifted to higher values by the presence of hybrid nanofillers. Finally, the flammability of the compositions was assessed. Flammability as reflected by the limiting oxygen index (OI) was increased by the presence of HNT and ATH-sil nanofiller and a hybrid system of the nanoparticles.

## 1. Introduction

The flammability of polymers has been recognized as a social and scientific problem for the wire and cable industry [1,2]. There are various types of polymers, which have been applied in the cable industry. Ethylene-vinyl acetate (EVA) copolymers and low-density polyethylene (LDPE) [3,4] have been extensively used in the wire and cable industry as excellent insulating materials owing to good mechanical and thermal properties. To improve the flame retardancy of these polymers, flame retardant agents may be added. Although the use of halogen-containing flame retardants has been eliminated due to their environmental danger [5], halogen-free flame retardant agents have been widely used to enhance flame retardancy [6,7,8]. Metal hydroxides, metal borates, organic phosphorus-based flame retardants, nitrogen-based flame retardants, and intumescent flame retardants are some examples of halogen-free flame retardant agents [5]. In addition, boron compounds, metal hydroxides, melamine (MLM), ammonium polyphosphate (APP), and pentaerythritol (PER) have been applied as flame retardant agents to modify the flame resistance of polymers [9]. Mineral filler fire retardants such as alumina trihydrate (ATH) and magnesium hydroxide (MH) are important fire-safe materials, especially for use in the cable industry [10]. ATH is colorless, inexpensive, and has good fire-retardant properties. ATH is a non-halogen-based material that can find application in flame retardation and smoke suppression [11]. ATH has been combined with polymers to improve flame retardancy [12,13]. However, to avoid incompatibility of the mineral filler with a polymer matrix, and thus obtain significant improvement in properties, one can find many publications confirming the effectiveness of the surface modification process of nanofillers, which includes the introduction of a coupling agent in the form of a silane [14,15,16]. It was confirmed that silane-treated ATH leads to a decrement in viscosity that affects the dispersion state of fillers in the matrix [14]. In this case, the fillers were well dispersed in the matrix in the presence of silane-treated ATH. Another naturally occurring mineral nanofiller is halloysite nanotubes (HNTs), with a chemical formula of Al_2_Si_2_O_5_(OH)_4_∙2H_2_O, with a high amount of 1D nanotubular structures exhibiting a high length-to-diameter ratio and low hydroxyl group density at the surface [17]. HNTs have been used in applications where high mechanical properties, thermal stability, and flame retardancy are desired. HNTs and ATH have been added to the polymers due to their high mechanical, thermal, and flame retardant properties. Halloysite nanotubes have been used to prepare a novel intumescent flame retardant (IFR) poly(butylene succinate) with anti-dripping properties [18]. HNTs and IFR filled (80 wt.%/20 wt.%) polypropylene (PP)/acrylonitrile butadiene styrene (PP/ABS) blends have been prepared using a twin-screw extruder [19]. Tensile and impact properties as well as thermal stability and flame retardancy increased with the addition of HNTs and to the PP/ABS blend. Zhao et al. [20] investigated the synergistic flame-retardant effect of halloysite nanotubes on IFR in LDPE. The results revealed that the addition of 2 wt.% of HNTs into LDPE/IFR improves the flame retardancy of the polymer [20]. Moreover, there have been many attempts to study the effect of flame retardant agents on the flame resistance of LDPE. Among them is a study by Shen et al. [21], who proposed a new strategy that blends LDPE with magnesium hydroxide (MH) and lauryl acrylate by electron-beam radiation. This composite has been registered in applications where high flame retardancy is desired. Qin et al. [22] investigated the effect of the addition of tris(2-hydroxyethyl) isocyanurate (THEIC) and silica-gel microencapsulated ammonium polyphosphate (MCAPP) on the flame retardancy and thermal properties of LDPE. The peak heat release and total heat release of the composite were remarkably decreased, and the thermal stability of the composites at high temperature was dramatically increased compared to that of neat LDPE [22]. Haurie et al. [23] have studied the effect of the addition of montmorillonite (MMT) and alumina hydroxide (ATH) as flame retardant systems into LDPE. The flame retardancy, thermal stability, and mechanical properties of LDPE improved in the presence of the MMT nanoclay [23]. Feng et al. [24] proposed an LDPE-based composite containing 4A zeolite. The introduction of 4A zeolite into the IFR system showed an obvious synergistic effect in the flame retardancy of LDPE. Chang et al. [25] introduced a flame retardant system composed of nano-kaolin and nano-sized hydroxyl aluminum oxalate (HAO) combined with LDPE/ethylene propylene diene rubber (EPDM) blends. The results revealed that the nano-kaolin had a synergistic effect with nano-HAO on flame retardancy in the LDPE/EPDM system [25]. Cao et al. [26] studied a zinc chelate complex containing both phosphorus and nitrogen for improving the flame retardancy of LDPE. The results showed that the flame retardant system could significantly increase the thermal stability, char formation, and smoke suppression ability of LDPE [26]. Lu et al. [27] investigated the effect of IFR and clay localization on the flame retardancy of linear LDPE/nylon 6 blends. The results indicated that the flame retardancy in the blends with clay localization in the LDPE phase was better than that of the blends with clay localization in the nylon 6 phase. Xu et al. [28] synthesized a novel IFR LDPE composite based on SiO_2_@MAPP and double pentaerythritol. The results revealed that the novel IFR polymer composites exhibit significant improvement in flame retardancy [28]. Wu et al. [29] studied the flame retardancy of LDPE with incorporated ultrafine zinc borate (UZB) and IFR. The flame retardancy of the polymer was improved with the addition of UZB to the LDPE/IFR system.

Recently there have been several studies on the effect of a hybrid system of nanofillers on the flame retardancy of polymers. Pandey et al. [30] studied the combined effect of multi-walled carbon nanotube (MWCNT) and organoclay on the flame retardancy and thermal stability of PP. Increased thermal stability and flame retardancy parameters for ternary nanocomposites were evident from thermogravimetric and cone calorimeter analyses. Moreover, layered double hydroxides and oxidized carbon nanotube as highly efficient flame retardant nanofillers have been added into PP to study the flame retardancy, thermal stability, and mechanical properties of the nanocomposites [31]. Feng et al. [32] studied the multiple synergistic effects of hexagonal boron nitride and flame retardant functionalized graphene on the thermal conductivity and flame retardancy of resin. The binary filler revealed an improvement in the thermal conductivity and flame retardancy of epoxy. Huang et al. [33] prepared novel organic–inorganic hybrid nanoparticles (phosphoryl polyethyleneimine amide–layered double hydroxide) to improve the smoke suppression and flame retardancy of thermoplastic polyurethane.

The influence of a hybrid system of nanofillers (halloysite nanotubes and clay) on the mechanical and thermal properties of EVA/LDPE intended for the cable industry was examined. The effects of HNTs in combination with silane-treated ATH on the flame retardancy of EVA/LDPE were studied.

## 2. Materials and Methods

### 2.1. Materials

In order to prepare samples for experiments, the following components were applied: a mixture of two ethylene-vinyl acetate (EVA) copolymers differing in the content of vinyl acetate (VA): ELVAX 260 (VA content = 28%, melt flow index (MFI) 5.0 g/10 min; density = 0.955 g/cm^3^) and ELVAX 40L-03 (VA content = 40%, MFI 3.0 g/10 min; density = 0.967 g/cm^3^) (Dupont, Wilmington, DE, USA); linear low-density polyethylene (LLDPE 1004, Exxon Mobil, Irving, TX, USA), with a density of 0.918 g/cm^3^ and MFI of 2.8 g/10 min (190 °C/2.16 kg); thermal stabilizer; modified EVA copolymer (coupling agent); plasticizer affecting the improvement of plasticity during extrusion; and stearin (Stearin 4963, Brenntag, Essen, Germany). The main component of each of the mixtures, equal to 60 wt.% in the reference sample and correspondingly less in polymer compositions (48 to 56 wt.%), was alumina hydroxide (ATH) (APYRAL 40CD, Nabaltec, Schwandorf, Germany).

In the analyzed compositions, at the expense of ATH, one added 4 and 8 wt.% of HNT (samples were designated as 4% HNT and 8% HNT, respectively), 4 and 8 wt.% of ATH-sil (the samples designated as 4% ATH-sil and 8% ATH-sil, respectively), and in two series of hybrids differing in the ratio of nanofillers, respectively: (i) at the mutual ratio of 1:1, 4 wt.% in total (2% + 2%, Hybrid_4% (1:1)) and 8 wt.% nanoparticles (4% + 4%, Hybrid_8% (1:1)); (ii) in hybrids with a mutual ratio of 2: 1, a total of 6 wt.% nanoparticles (4% + 2%, Hybrid_6% (2:1)) and 12 wt.% of nanoparticles (8% + 4%, Hybrid_12% (2:1)).

HNT (under the trademark “Dunino”, Intermark (Gliwice, Poland)) was used in the form of a powder with a bulk density of 450–600 g/dm^3^ containing both rod-shaped and flake-shaped halloysite nanoparticles, which are loosely bound to each other by weak van der Waals bonds. However (from the producer data), since most of the material consisted of rod-shaped nanoparticles with a diameter of 100–140 nm and length of 1–5 μm, authors marked the material as HNTs (halloysite nanotubes).

The second nanofiller was surface-modified alumina trihydrate (marked as “ATH-sil”), APYRAL 40 VS1 (Nabaltec, Schwandorf, Germany), vinyl-silane-treated mineral flame retardants for the wire and cable industry with 98.5% content of Al(OH)_3_, specific surface area of 3.5 m^2^/g, bulk density of 350 kg/m^3^, and density of 2.4 g/cm^3^.

**Figure 1:**
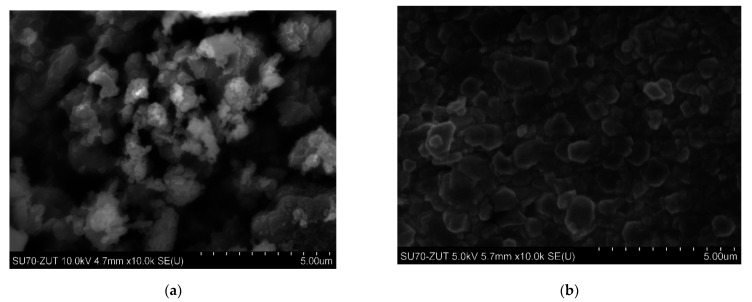
Scanning electron micrographs of nanofillers used in the study: (**a**) HNTs (as received); (**b**) ATH-sil (as received).

Figure 1a,b shows the scanning electron microscopy (SEM) images of both nanofillers (as received): HNTs and ATH-sil at the same magnification of 10k. While the composition of the prepared materials is presented in Table 1.

### 2.2. Preparation of the Materials and Testing Samples

The series of materials were prepared on a counter-rotating twin-screw extruder (LSM30, Leistritz Laborextruder, Cottbus, Germany) with closely overlapping coils and interchangeable mixing sections (diameter D = 34 mm, L/D ratio = 23), following the same procedure as previously published in [34].

The specimens for determining the mechanical properties and hydrostatic density were made by injection molding using a Boy 15 (Dr. Boy, Neustadt-Fernthal, Germany) injection molding machine with a mold clamping force of 150 kN. The dimensions of the measuring part of the dumbbell-shaped sample corresponded to the standardized dimensions (sample type 3) according to the PN-ISO 37 standard. The parameters of the injection process were selected on the basis of the PN-EN ISO 294 standard, and the melting point of the material was determined based on differential scanning calorimetry (DSC) measurements. Before performing the measurements, the samples were conditioned in accordance with the recommendations of the PN-EN ISO 291 + AC1 standard.

The samples for the flammability and OI tests, as well as the hardness measurements, were prepared by pressing using a hydraulic press (P 200 E plate press, Dr. Collin GmbH, Maitenbeth, Germany). The materials were pressed at a temperature of 170 °C, each time using 4 mm thick spacers.

Samples for absorbency tests in the form of strips with a standard size of 80 mm × 5 mm and a thickness of 0.8 mm were cut from the moldings in the form of sheets prepared on a hydraulic press.

### 2.3. Characterization Methods of Polymer Compositions

The morphology of the polymer (hybrid) compositions was characterized by scanning electron microscopy (SEM, Hitachi SU-70, Naka, Japan) with an accelerating voltage of 5 kV and a working distance of 12 mm. The samples for SEM analysis were cryofractured in liquid nitrogen and subsequently coated (2–5 nm) in a vacuum with a thin gold film before the tests. Moreover, for hybrid nanocomposite containing the total highest amount of nanofillers (Hybrid_12% (2:1) the energy dispersive spectrum (EDS) and mapping were performed in order to identify the contributing elements through their characteristic radiation, collected from a selected area. The estimated weight concentrations of all detected elements were quantified, expressed as weight percentage (wt.%), and tabulated along with EDS spectra, which show the relative concentration of an element in the analyzed area.

The static mechanical properties of the polymer (hybrid) compositions were measured on a universal tensile test machine (Autograph AG-X plus, Shimadzu, Kyoto, Japan) equipped with an optical extensometer at room temperature according to PN-ISO 37 and PN-EN ISO 527. The stress–strain curves were obtained at a strain rate of 250 mm/min. For each material, five to ten measurements were performed, whereas for values not different from each other by more than 5%, the results were averaged.

Hardness tests were determined by the Shore method according to a standard EN ISO 868:2003 on a Zwick 3100 Shore D tester (Zwick GmbH, Ulm, Germany).

The absorption tests were carried out in accordance with the standard IEC 60502-2 according to the procedure described in the standard IEC 60811-402, whereas the details of the procedure can be found in our open-access paper [35].

The melt flow index (MFI) measurements were performed using a melt flow tester (CEAST, Pianezza TO, Italy) according to ISO 1133 standard at 150 °C and under a load of 2.16 kg.

The Vicat softening temperature (VST) was measured by Vicat apparatus (VEB Thüringer Industriewerk Rauenstein, Germany) according to ISO 306:2013 standard. The tests were carried out following the A50 protocol, i.e., using a force of 10 N and heating rate of 50 °C/h. The VST was measured as the temperature at which the indenting tip penetrated to a depth of 1 mm into the specimen. Each reported value is the mean of at least three independent measurements.

Differential scanning calorimetry (DSC) measurements were performed on a DSC 204 F1 Phoenix (Netzsch, Selb, Germany). Samples with the mass of approximately 10 mg were encapsulated in an aluminum crucible (pan) and then heated from −75 to 250 °C at a scan rate of 10 °C/min and held at 250 °C for 5 min to melt the samples and to erase thermal history, before cooling. Subsequently, the samples were cooled to −75 °C and again heated (second heating scan) to 250 °C using the same scan rate. The first cooling and second heating scans were used to determine the melting and crystallization peaks.

The thermo-oxidative stability of ATH, ATH-sil, and polymer compositions was studied by using thermogravimetric analysis (TGA 92-16.18 Setaram, Caluire-et-Cuire, France). Measurements were carried out in an oxidizing atmosphere, i.e., dry, synthetic air (N_2_:O_2_ = 80:20 vol.%) at a flow rate of 20 mL/min. The study was in the temperature range of 20–700 °C at a heating rate of 10 °C/min. The flammability of the materials was evaluated using the OI method according to the procedure described in the EN-ISO 4589-2 standard.

## 3. Results

### 3.1. Dispersion Characteristics

**Figure 2:**
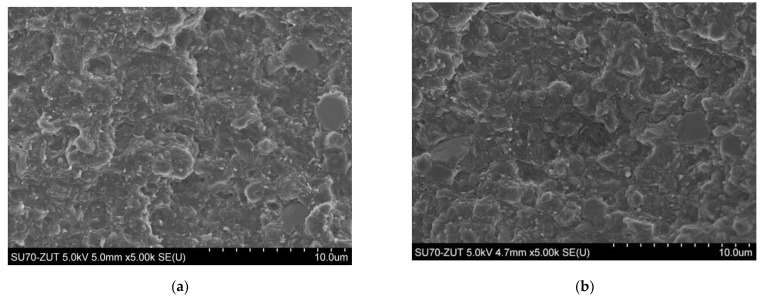

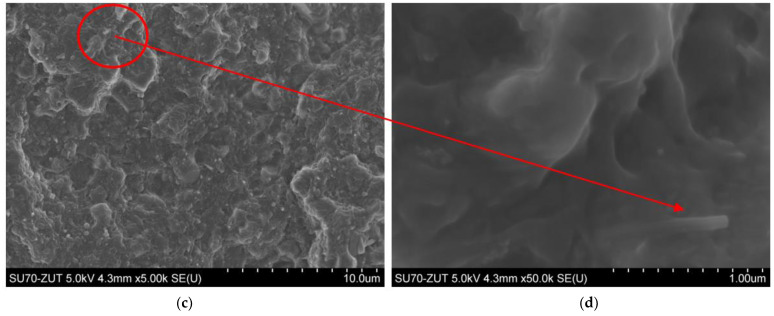
Scanning electron micrographs of (**a**) nanocomposite with 8% of HNTs (8HNT); (**b**) nanocomposite with 8% of ATH-sil (8ATH-sil); (**c**,**d**) hybrid nanocomposite with 12% of nanofillers (2:1) HNTs and ATH-sil (H12 2:1).

SEM micrographs prepared for the two compositions with the highest proportion of nanofillers, i.e., 8 wt.% HNT and ATH-sil, respectively (Figure 2a,b), show a polymer matrix with well-dispersed particles (in the forms of tubes and platelets). However, microscopic analysis of the fracture surfaces of the samples shows the presence of single agglomerates (inclusions) from both types of nanofillers. A few nanotubes are visible, protruding from the polymer matrix. All tested samples show a partially crystalline structure, which proves a good dispersion of the fillers, and will be further confirmed by DSC analysis.

Silane-modified ATH (ATH-sil) is an environmentally friendly, halogen-free, non-toxic, non-aggressive, non-volatile, inert, recyclable, finely precipitated aluminum hydroxide [36]. It can be applied as a flame retardant agent and as a filler in powder coatings, paints, varnishes, and plasters [14]. It also offers a reduced smoke density and does not affect pigmentation. ATH (APYRAL 40CD), used to prepare the reference material, had an unmodified surface, which could lead to a limited “anchoring” of the filler particles in the polymer matrix, which led to a deterioration of mechanical properties and even reduction of flame resistance (filler agglomeration). This is especially important for highly filled materials (approx. 60% filling), just as it is in our reference materials. To ensure an increase in the strength of the highly filled materials, the surface of the fillers should be modified. The ATH manufacturer (Nabaltec) performed the surface modification process, using methylsilane. Silanization of the surface of mineral filler particles (such as ATH) aims to improve their compatibility with the polymer matrix by increasing interfacial interactions. This has a direct impact on the mechanical properties of the material (the use of silanes as “coupling agents” results in a favorable increase in tensile strength).

Halloysite differs from other fillers such as silica, calcium carbonate, kaolin, or montmorillonite due to its nanometric plate-and-tube structure [37,38]. Halloysite materials exhibit compact block structure and strong internal bonds; therefore, only the surface of the grains interacts with the polymer matrix. Thus, halloysite will fulfill its role only with good deagglomeration of the grains and their even distribution in the matrix (hence the preparation of a hybrid with both a 1:1 and 2:1 proportion, which may contribute to the improvement of the dispersion quality). Such a condition will ensure an increase in the strength of the material filled with HNT and an improvement in thermal parameters and flame resistance (the improvement in fire resistance has been confirmed many times in the prepared mixtures containing HNT [39]).

**Figure 3:**
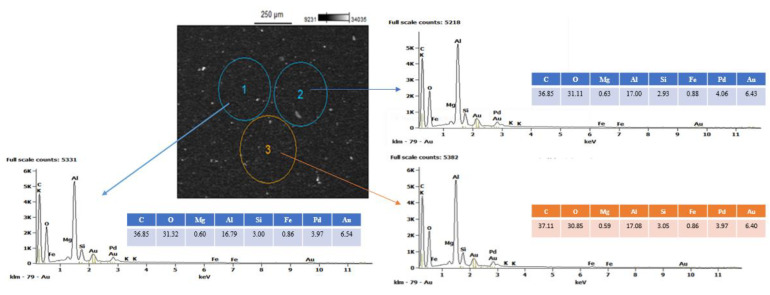
Microanalysis SEM/EDS along with the area EDS spectra (at three different locations (2:1) HNTs and ATH-sil.

The dispersion evaluation carried out in hybrid systems containing 12 wt.% of HNT and ATH-sil (H12%, 2: 1), made on the basis of the SEM analysis of the fractured sample, confirms the homogeneity of the material and, thus, good dispersion of fillers (Figure 3). Moreover, in the SEM image for this composition, at two different magnifications (Figure 2c,d), individual grains with size from a few to 20–30 µm can be seen, the composition of which indicates that they may be grains of halloysite agglomerates or iron compounds, but at higher magnification, “pull-outs” of nanofibers from the polymer matrix, formed during the fracture, are visible, which indicates that the copolymer forms a continuous phase in the mixture (indicated with the arrow, Figure 2d).

**Figure 4:**
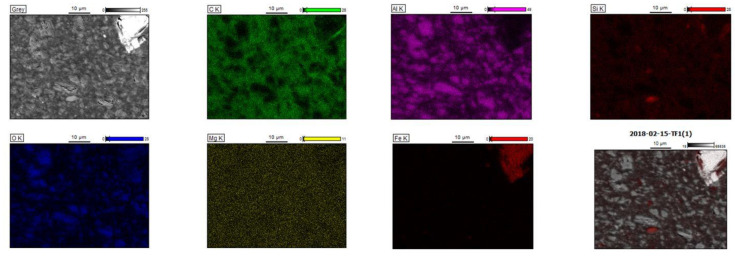
SEM/EDS base image and elemental mapping for C, carbon; Al, aluminum; Si, silica; O, oxygen; Mg, magnesium; and Fe, ferrite of the hybrid nanocomposite containing 12% (2:1) HNTs and ATH-sil.

Similar conclusions could be drawn from the study of the local elementary composition by the SEM/EDS method. EDS microanalysis allows for the identification (surface and volume) of the chemical elements included in the tested material. EDS analysis can be both qualitative and quantitative. EDS microanalysis, performed three times (marked in Figure 3 with circles) for the material with the highest content of nanoparticles (Hybrid_12%), shows homogeneity of composition and good dispersion of both nanofillers. The elemental composition is comparable in the entire analyzed sample volume: the differences between the content of individual elements are insignificant. Moreover, the presence of mineral nanoparticles (presence of Si) was clearly demonstrated, but so also was the fact that HNT contained a certain percentage of Fe. Additionally, elemental mapping (Figure 4) for C, carbon; Al, aluminum; Si, silica; O, oxygen; Mg, magnesium; and Fe, ferrite of the hybrid nanocomposite containing 12% (2:1) HNTs and ATH-sil, also clearly confirmed the homogeneity of the composition and good dispersion of both fillers.

### 3.2. Mechanical and Processing Performance

**Figure 5:**
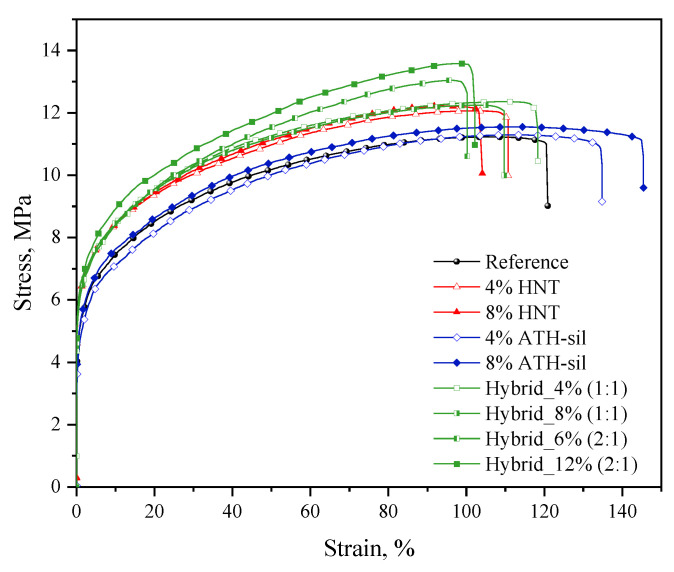
Representative stress–strain curves for the series of polymer compositions containing HNTs and/or ATH-sil.

As can be seen from the performed measurements, the mechanical and processing parameters vary considerably depending on the amount and type of flame retardant agent. In this work, it was noted that, in order to improve the mechanical performance of the flame retardant compositions, the bond strength of the matrix with the filler surface is of great importance. In the considered polymer compositions, the matrix mainly consists of an LDPE/EVA copolymer. Polyolefins such as polyethylene have a low polarity, which makes it practically impossible to form bonds with polar halloysite crystals [40,41]. This prevents the polymer from penetrating the pores and the so-called galleries of layered aluminosilicates used in mixtures. On the other hand, EVA is a highly polar copolymer known to form strong bonds with polar fillers [38]. In the case of a PE/EVA blend, the mere presence of EVA may not be sufficient to form strong bonds with the polar filler. This can be achieved by an appropriate blending process, taking into account the characteristics of the individual components or by using appropriate compatibilizers. In this case, the compatibilizer improves the interaction between the polar filler and the matrix and also improves the dispersion of the filler therein. For this purpose, ATH-sil was used not only as a flame retardant agent but also as a compatibilizer that improves mechanical and processing performance in the considered systems (especially hybrids). It should also be noted that HNT (Dunino) is characterized by a high specific surface area; easy access to active hydroxyl groups on the developed surface of the nanoplates (in the tubes they are inside them and are difficult to access); a diversified zeta potential on the surface silanol (negative) and aluminol (positive), which significantly extends the possibilities of creating bonds with polar polymers or with surface modifiers in the case of non-polar polymers; and a large number of active centers resulting from substitutions for Al and Si in the crystal structure and weak bonds between lamellar and tubular single crystals, facilitating grain disaggregation in the matrix [17,42]. Figure 5 represents the stress–strain curves for the EVA/LDPE blend and its compositions reinforced with HNT and ATH-sil nanofillers. The tensile strength increased in the presence of HNT; however, the elongation at break decreased with the addition of HNT nanofillers into the blend, as presented in Table 2. This reduction in E_b_ is due to the presence of the agglomerated particles in the matrix [43]. In the case of ATH-sil (4% and 8%) nanofillers, the tensile strength slightly increased, while the elongation at break significantly improved compared with that of the reference sample. In the case of the hybrid nanocomposite, the tensile strength increased, however, the elongation at break for the hybrid compositions decreased. The HNT and ATH-sil nanofillers created a hybrid nanocomposite with superior mechanical properties. Moreover, the hardness of the reference sample increased with the addition of HNT, ATH-sil, and a hybrid system of those nanofillers. This increment is due to the fact that the nanofillers play a significant role in the hardness properties of the nanocomposites. The water absorption of EVA-LDPE decreased along with the increase in the content of HNT and ATH-sil. Finally, the MFI of the reference increases with an increase in the amount of nanofillers. This shows that samples flow better even with a high content of nanofillers.

### 3.3. Thermal Properties and Flame Retardancy

**Figure 6:**
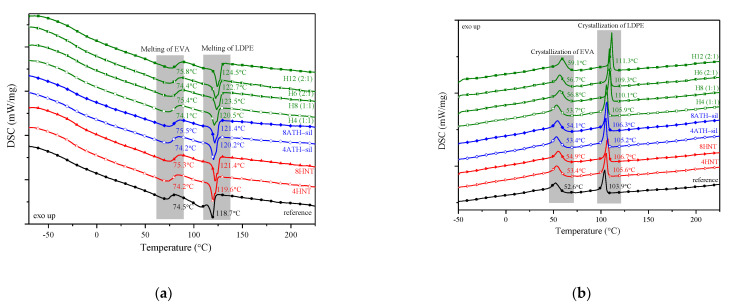
Differential scanning calorimetry (DSC) thermograms recorded during (**a**) 2nd heating and (**b**) cooling for the series of compositions containing HNTs and/or ATH–sil.

Differential scanning calorimetry, Vicat softening temperature, and thermal degradation analysis were applied to study the thermal properties of the reference (EVA/LDPE blend) and its polymer compositions reinforced with HNTs and ATH-sil nanofillers. Figure 6 shows the DSC curves for the specimens. Moreover, the values of Vicat softening temperature, melting temperature (T_m_), melting enthalpy (ΔH_m_), crystallization temperature (T_c_), and crystallization enthalpy (ΔH_c_) are summarized in Table 3. There are no significant differences in the values of VST in the presence of HNTs and ATH-sil nanofillers. Moreover, the melting temperature of the reference sample was shifted to a higher temperature on the addition of HNT and ATH-sil nanofillers. The values of crystallization temperatures as well as corresponding crystallization enthalpies were also affected by the incorporation of both nanofillers, which is especially visible for the highest content of nanofillers (Hybrid_12%).

**Figure 7:**
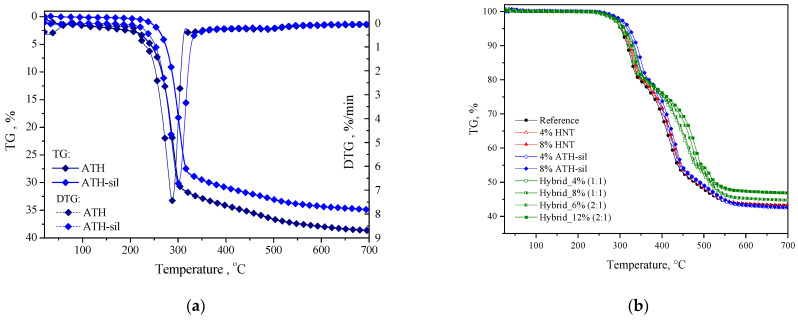
Thermal degradation behavior evaluated in the oxidizing atmosphere for (**a**) ATH and ATH-sil and (**b**) prepared materials.

On TGA curves for pristine ATH and ATH-sil (Figure 7a), one can observe clear differences. The detailed analysis on these two materials can be found in our previously published paper [35], but in brief: pristine ATH exhibited two mass losses: the first one in the temperature range of 20–165 °C corresponds to the loss of adsorbed water [14] and the second major mass loss in the temperature range of 180–600 °C corresponds to the decomposition of surfactant molecules [44]. In turn, the ATH-sil decomposes in one major step, however, its decomposition temperature was improved by about 20 °C. It is especially well observed at the DTG, where the maximum of thermal decomposition has been shifted from 288 °C (pristine ATH) to 300 °C for the ATH-sil. Such improvement in thermo-oxidative stability confirms the legitimacy of using ATH-sil, as it is well known that such surface treatment affects the dispersibility of the filler and the water absorption of the final material and improves the mechanical properties [45].

Figure 7b shows the mass loss and derivative of mass loss for the prepared compositions. Furthermore, Table 3 presents the temperatures related to the 5% and 50% mass loss for the samples. The reference represents a mass loss of 5% at 297 °C, as shown in Table 3. The thermal stability of the reference material was slightly improved in the presence of 8% of HNT nanofillers. The presence of ATH-sil significantly affected the thermo-oxidative stability of PE/EVA blend. The degradation temperature increased from 297 to 318 °C on the addition of 8% of ATH-sil into the reference. The strong effect of ATH-sil on the reference is due to the modification of the surface of ATH with layered silicates. In addition, the thermal stability of the reference was shifted to higher temperatures in the presence of the hybrid system of nanoparticles. This improvement is due to the high thermal stability of HNTs and ATH-sil nanofillers that were added to the reference polymer. However, the ATH-sil represented a significant role in the improvement of the thermal stability of the hybrid systems compared with the HNT nanoparticles. Finally, the thermal stability of the reference was improved from 467 to 520 °C at the temperature of 50% mass loss in the presence of the hybrid system of nanofillers (8% HNT, 4% ATH-sil). An important aspect is the predominance of lamellar crystals compared to tubular ones, which makes it possible to form more bonds on both the silane and the aluminol sides. It also allows obtaining a larger blockage area for the flow of oxygen and heat, which is important for increasing the fire resistance of the materials and improving their thermal indicators [17,42].

**Figure 8:**
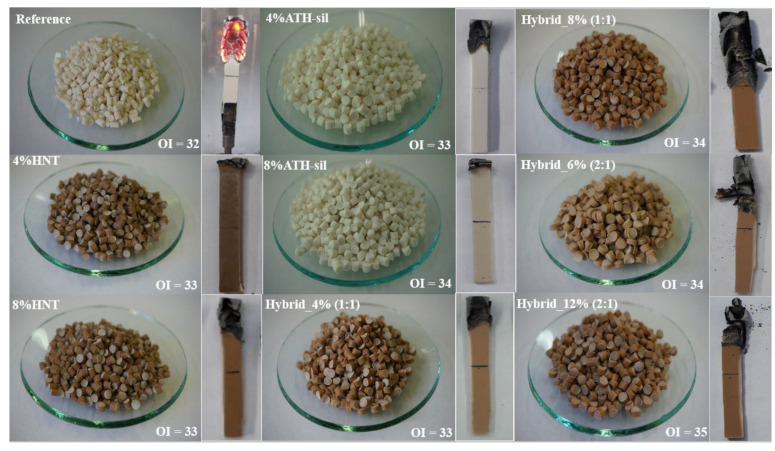
The appearance of the prepared granulates and samples after the flammability test.

The flame retardancy measurement (OI values with photos of the granulates and samples subjected to the test) of the reference and its nanocomposite is shown in Figure 8. The flame retardancy of the samples has been measured according to the EN-ISO 4589-2 standard. According to the standard, the flame retardancy of the specimens has been classified as follows: (1) oxygen index (OI) below 21%—strongly combustible material; (2) OI within the range of 21–28%—combustible material, and (3) OI above 28%—flame retardant (self-extinguishing) material. Herein, all samples showed an OI above 28% and the samples were self-extinguishing. In each case of ignition, the time criterion was exceeded (the sample burned for more than 180 s). In the case of the sample containing HNT and ATH-sil nanofillers, the burnt sample length criterion was not exceeded, and the flame advanced very slowly. In the case of hybrid systems, the burnt lengths extended till the middle of the sample; however, the OI increased to higher values. Moreover, the reference sample and compositions containing ATH-sil were white. However, the sample containing HNT caused a change in color into brown. It is known that ATH decomposes via an environmentally friendly mechanism known as dehydration (ATH releases water molecules at 220 °C). This endothermic reaction results in the formation of two non-toxic ingredients—aluminum oxide (Al_2_O_3_), which forms an inert residue, and water, which dilutes the smoke [14,15,46,47]. The decomposition products, as already mentioned above, lower the temperature and dilute the combustible gases. However, the effectiveness of these compounds is not as great as that of fluorine compounds. Therefore, one can modify the sample surface (e.g., silane modification) or mix it with other nanofiller in order to obtain a hybrid system of fillers with a positive hybrid effect on improving thermal stability and flame retardancy. HNTs are such a nanofiller that could significantly enhance the compactness and thermal stability of the barrier layer and improve the flame retardancy efficiency of different polymer matrices [17,18,19,42]. The barrier layer covering the polymer matrix surface could effectively cut off the heat and material exchange between the polymer matrix and the combustion flame zone and prevent further degradation [48]. Such environmentally friendly decomposition of polymer composition containing halogen-free fillers intended for the cable industry is of great interest to both scientific and industrial applications.

## 4. Conclusions

The HNTs and ATH-sil nanofillers were added into the EVA/LDPE polymer blend (reference sample) to prepare a high-flame-retardance nanocomposite that can find application in the cable industry. The morphology of the polymer compositions was studied via SEM, and the results indicated that the nanofillers were homogeneously dispersed in the reference sample even at higher content of nanofillers in the case of the hybrid systems. The tensile properties were improved along with the addition of HNTs and ATH-sil nanofillers into the blend. The hybrid samples showed significant changes in tensile strength. Moreover, the melting temperature and crystallization temperature increased in the presence of HNTs and ATH-sil nanofillers. The degradation temperatures of the materials were enhanced with the addition of both types of nanoparticles. The flame retardancy and the OI of the blend were improved along with the addition of HNTs, ATH-sil, and a hybrid system of nanofillers.

## Figures and Tables

**Table 1:** Compositions of the prepared mixtures.

Sample	EVA (%)		LLDPE (%)	Coupling Agent (%)	Plasticizer (%)	ATH Apyral(%)	Nanofillers		Thermal Stabilizer (%)	Stearin (%)
	ELVAX 260	ELVAX 40L					HNT (%)	ATH-sil (%)		
Reference	20.8	4	7.5	5	2	60	-	-	0.2	0.5
4%HNT	20.8	4	7.5	5	2	56	4	-	0.2	0.5
8%HNT	20.8	4	7.5	5	2	52	8	-	0.2	0.5
4%ATH-sil	20.8	4	7.5	5	2	56	-	4	0.2	0.5
8%ATH-sil	20.8	4	7.5	5	2	52	-	8	0.2	0.5
Hybrid_4% (1:1)	20.8	4	7.5	5	2	56	2	2	0.2	0.5
Hybrid_8% (1:1)	20.8	4	7.5	5	2	52	4	4	0.2	0.5
Hybrid_6% (2:1)	20.8	4	7.5	5	2	54	4	2	0.2	0.5
Hybrid_12% (2:1)	20.8	4	7.5	5	2	48	8	4	0.2	0.5

**Table 2:** Mechanical properties, hardness, water absorption, and MFI of EVA/LDPE and its nanocomposites.

Sample	TS (MPa)	E_b_ (%)	H (ShD)	Water Absorption (%)	MFI (21.6 kg/150 °C)
Reference	11.22 ± 0.20	120.17 ± 5.71	38 ± 1	2.46	5.3
4%HNT	12.06 ± 0.18	110.22 ± 7.35	41 ± 1	2.22	5.3
8%HNT	12.23 ± 0.32	103.23 ± 5.54	41 ± 1	2.05	5.6
4%ATH-sil	11.29 ± 0.17	134.12 ± 9.16	43 ± 1	2.17	5.5
8%ATH-sil	11.55 ± 0.22	144.94 ± 11.38	44 ± 1	1.94	6.0
Hybrid_4% (1:1)	12.36 ± 0.13	117.80 ± 7.47	47 ± 1	1.92	5.9
Hybrid_8% (1:1)	12.25 ± 0.17	109.12 ± 7.24	51 ± 1	1.78	6.0
Hybrid_6% (2:1)	13.04 ± 0.23	110.24 ± 5.63	51 ± 1	1.26	6.1
Hybrid_12% (2:1)	13.38 ± 0.21	102.73 ± 4.85	54 ± 1	0.91	6.1

TS—tensile strength; E_b_—elongation at break; H—hardness; MFI—melt flow index.

**Table 3:** Thermal properties of EVA/LDPE (reference), and prepared compositions.

Sample	VST (°C)	T_m(LDPE)_ (°C)	ΔH_m_ (J/g)	T_c(LDPE)_ (°C)	ΔH_c_ (J/g)	T_5%_ (°C)	T_50%_ (°C)
Reference	60.5 ± 1.1	118.7	5.02	103.9	5.11	297	467
4%HNT	59.8 ± 0.4	119.6	5.78	105.6	5.54	302	472
8%HNT	60.4 ± 0.7	121.4	5.89	106.7	5.76	304	474
4%ATH-sil	59.2 ± 1.3	120.2	5.81	105.2	5.80	311	473
8%ATH-sil	60.2 ± 0.6	121.4	5.92	106.3	6.01	318	479
Hybrid_4% (1:1)	59.5 ± 0.6	120.5	5.84	105.9	5.79	302	506
Hybrid_8% (1:1)	61.5 ± 1.1	123.5	6.24	110.1	6.27	305	509
Hybrid_6% (2:1)	60.1 ± 1.0	122.7	6.12	109.3	6.03	297	513
Hybrid_12% (2:1)	61.5 ± 0.7	124.5	6.91	111.3	6.84	304	520

VST—Vicat softening temperature; T_m_—melting temperature and corresponding enthalpy of melting ΔH_m_; T_c_—crystallization temperature and corresponding enthalpy of crystallization ΔH_c_; T_5%_ and T_50%_—temperatures corresponding to 5% and 50% mass loss.

## Data Availability

The data presented in this study are available on request from the corresponding author. The data are not publicly available due to the fact that these are data on the research work carried out in the project, which lasts until July 2021. At the moment, the selected data for this project are only kept in open repositories.
